# Dataset of depressive posts in Russian language collected from social media

**DOI:** 10.1016/j.dib.2020.105195

**Published:** 2020-02-04

**Authors:** Sergazy Narynov, Daniyar Mukhtarkhanuly, Batyrkhan Omarov

**Affiliations:** a Alem Research, Almaty, Kazakhstan; b Suleyman Demirel University, Almaty, Kazakhstan; c International Information Technology University, Almaty, Kazakhstan; d Al-Farabi Kazakh National University, Almaty, Kazakhstan; e Khoja Akhmet Yassawi International Kazakh-Turkish University, Turkistan, Kazakhstan

**Keywords:** Depression, Dataset, Machine learning, NLP, Social network, Suicide

## Abstract

This paper presents dataset collected from social networks that are mostly used by youth of Commonwealth of Independent States (CIS) countries. The data was collected from public accounts of VKontakte social network by using VK.api and applying the most used keywords that would signify depressive mood. The collected data was classified by psychologists into two types: depressive and non-depressive. The dataset consists of 32 018 depressive posts and 32 021 non-depressive posts. Since the most common language that is spoken in CIS countries is Russian, the posts are written in Russian, consequently the collected data is in Russian language as well. The data can mostly be useful for researchers who explore tendencies to depression in CIS countries. The dataset is important for the research community, as it was not only collected from open sources, but also marked by our psychiatrists from the republican scientific and practical center of mental health. Since the dataset has very high validity, it can be used for further research in the field of mental health.

**Table:** Specifications Table

Subject	Artificial Intelligence, Natural Language Processing (NLP), Machine Learning
Specific subject area	Depressive post detection, depressive account detection, suicidal post detection, depressive content detection
Type of data	Table Excel file
How data were acquired	Data were collected from public accounts in social networks by using keywords and classified by psychologists into depressive and non-depressive categories. Instruments: software, program Model and make of the instruments used: classification into several categories by experts
Data format	Raw, Analyzed
Parameters for data collection	For data collection, a bot in Python was created that collects posts from social media public accounts by keywords that refer to depressive behaviour. List of keywords for data collection: suicide, I don't want to live, life is shit, I want to die, etc.
Description of data collection	The collected data were divided into two parts as depressive posts with label 1, and non-depressive posts with label 0
Data source location	Azerbaijan, Armenia, Belarus, Kazakhstan, Kyrgyzstan, Moldova, Russian Federation, Tajikistan, Ukraine, Uzbekistan
Data accessibility	With the article In a public repository: Repository name: https://data.mendeley.com/datasets/838dbcjpxb/1 Data identification number: https://doi.org/10.17632/838dbcjpxb.1 Direct URL to data: https://data.mendeley.com/datasets/838dbcjpxb/1/files/c88e7ab7-3dba-4a1b-8c83-6b78b8adb2a5/Depressive%20data.xlsx?dl=1

**Table:** 

**Value of the Data** • This dataset can be useful for researchers in the machine learning field to train neural networks in order to detect depressive and suicidal accounts in social networks. • The dataset can be useful for data scientists who do research in social, psychological and health informatics area as an analysed data • The dataset can be used to train and evaluate computational models and techniques for automation or to help experts identify depressive accounts to reduce suicides; • These data were collected from Vkontakte social networks, then analysed and filtered, that can be used by other researchers as a benchmark dataset in machine learning, and deep learning fields.

## 1 Data

We have sourced 64 039 posts in Russian language that contain depression-related keywords. About half of them (32 021 posts) are labelled as 0 which we would refer to as neutral posts, the other part (32 018 posts), which is labelled as 1, consists of depression-related posts.

Since the data on people who committed suicide is private, we collected depressive posts of social network users. We collected around 32 018 depressive posts and 32 021 usual posts such as news, blogs, advertisements, and etc.

In Table 1, we provide examples of depressive posts by indicating the age of authors.

**Table 1:** Samples of depressive posts.

	text	label	age
0	Когда-то я был добрым романтиком, который стре...	1	32.0
1	Здрастϑуйте! Я каждый день просыпаюсь с мыслью...	1	28.0
2	У меня проблемы с деϑу♯кой. Каждую ссору я не ...	1	16.0
3	Вся моя жизнь это один спло♯ной ад, ϑ котором ...	1	32.0
4	Я хочу уснуть и не проснуться.каждый день одно...	1	14.0

**Fig. 1:**
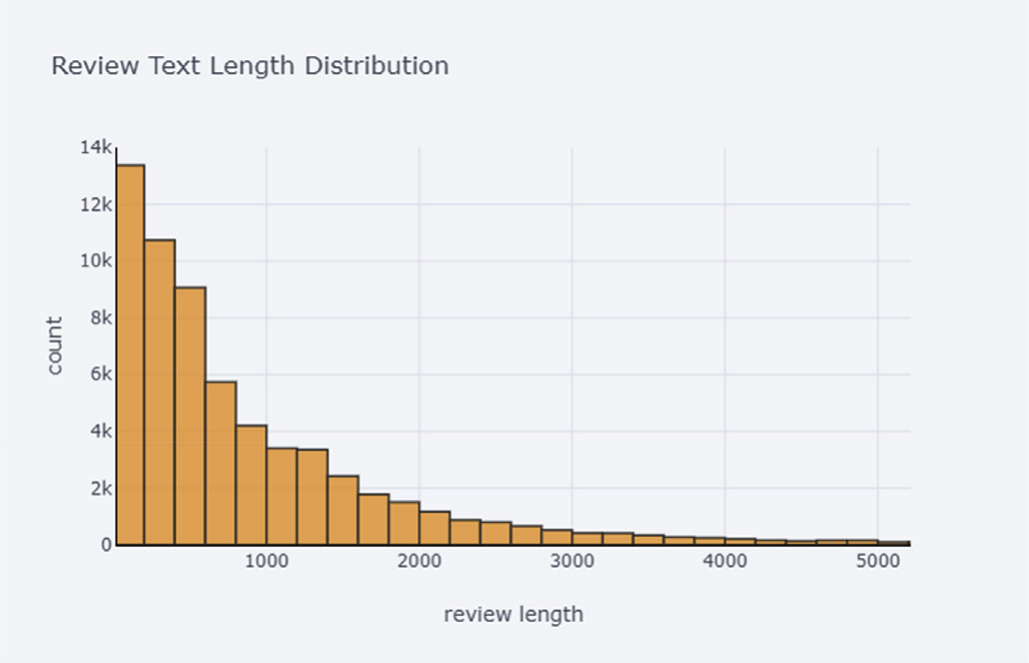
Text length distribution of posts.

Fig. 1 illustrates the text length distribution of posts. It can be noticed that depressive related posts are distributed according to the law of exponential distribution, which means many texts are of short length.

**Fig. 2:**
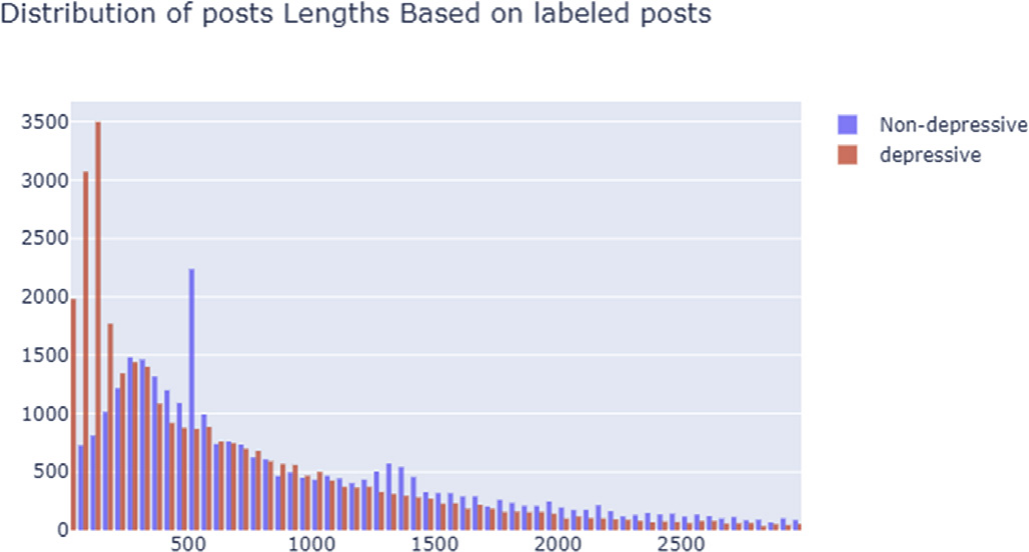
Distribution of posts lengths by labels.

Fig. 2 shows distribution of depressive and neutral posts in the dataset. Red line indicates depressive posts, Blue line indicates non-depressive posts.

**Fig. 3:**
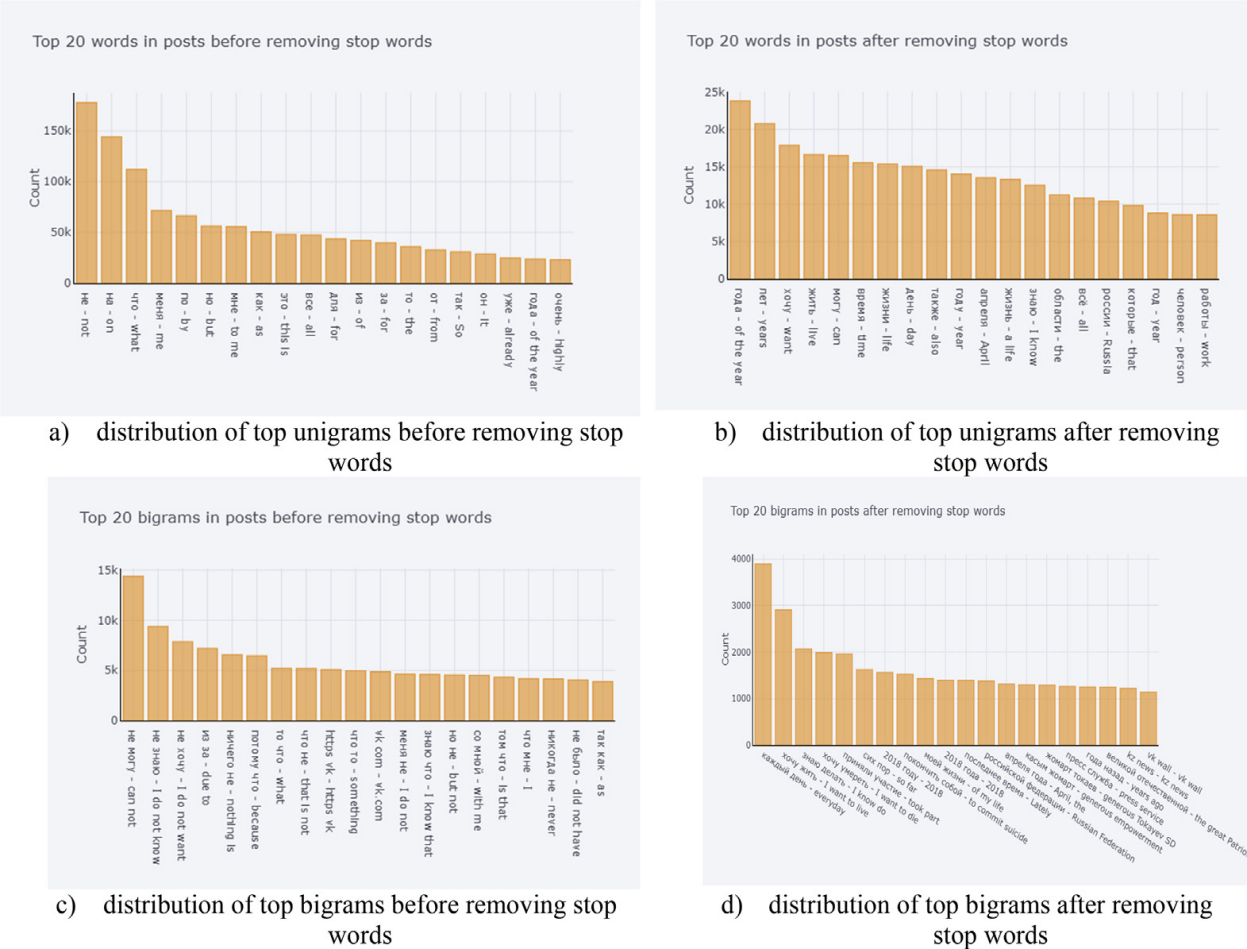
Distribution of top unigrams and bigrams.

In Fig. 3, we explore the data around the stop words as the stop words can significantly influence the meaning of the sentence. Fig. 3a and b illustrate the distribution of top 20 unigrams, taking into account the stop words, without considering the stop words, respectively. Fig. 3c and d demonstrate the distribution of bigrams with and without application of stop words, correspondingly.

**Fig. 4:**
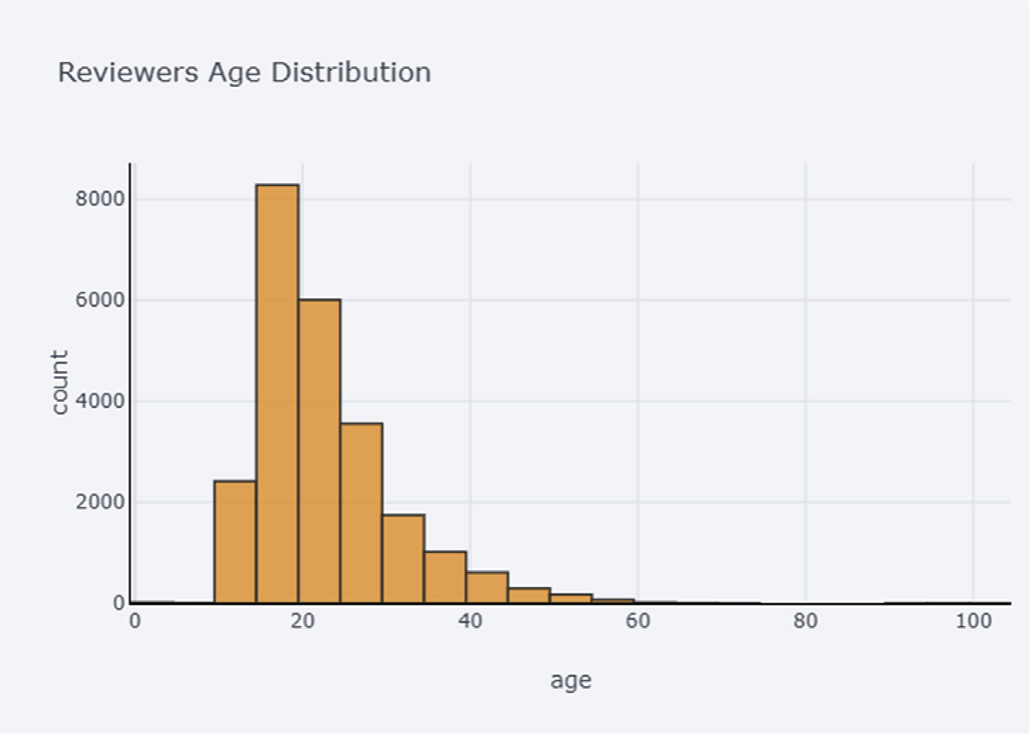
Age distribution.

Fig. 4 illustrates the age distribution of authors of depressive posts. The distribution spreads by the normal distribution with offset to the left side, which implies that people that are most prone to depression are teenagers and youth under 30 years.

**Fig. 5:**
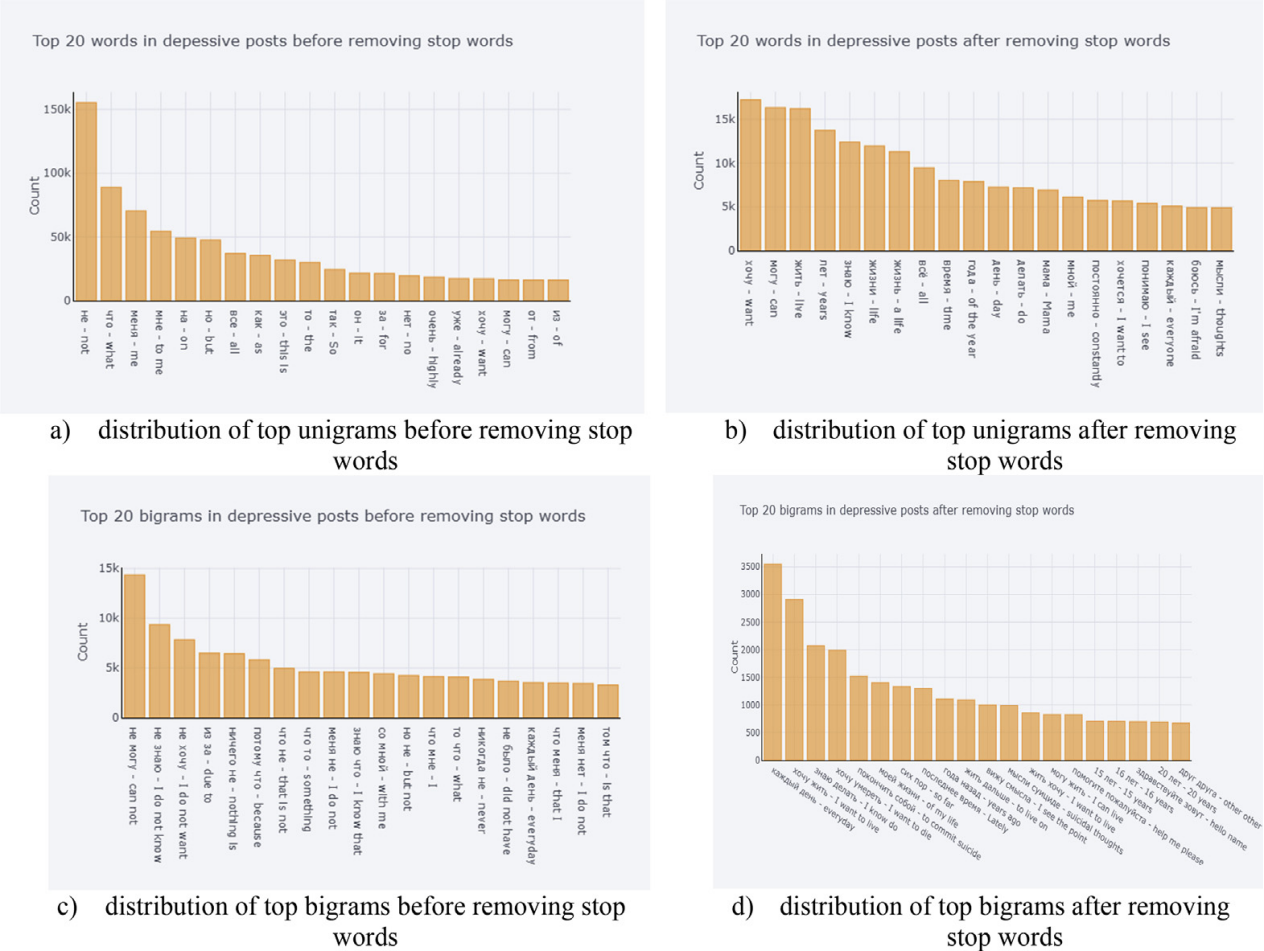
Distribution of top unigrams and bigrams taking into account only depressive posts.

Fig. 5 explores the depressive related data around the stop words. Fig. 5a and b illustrate the distribution of top 20 unigrams in the depressive posts, with and without taking into account the stop words, respectively. Fig. 5c and d display the distribution of bigrams in the depressive posts, with and without using the stop words, appropriately.

The given dataset can be useful to train a neural network in order to detect depressive related accounts in social media or other online resources.

## 2 Experimental design, materials, and methods

**Fig. 6:**
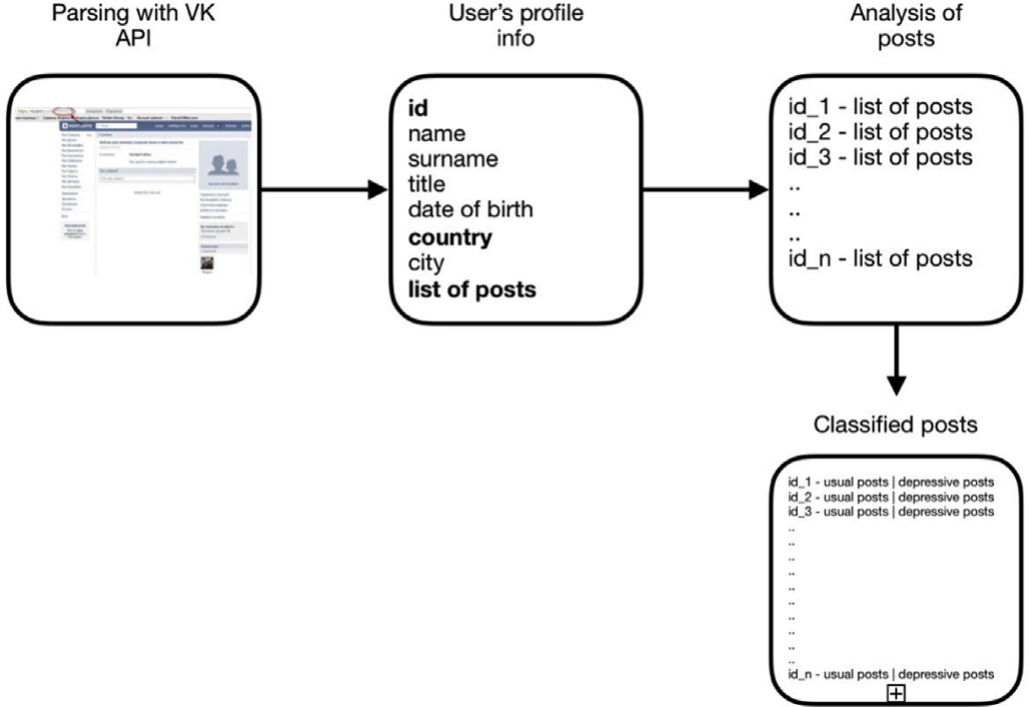
Scheme of data acquisition, analysis and classification of posts.

Before attributing information to suicidal or depressive, it is necessary to define the criteria of “danger”. One of the solutions is the definition of a set of keywords. It is this method of determining the types of information that was applied in the developed software package. For the definition a set of keywords was compiled, which was used to analyse information on the social network VKontakte [1]. The software package based on the presence or absence of the specified keywords in the text concludes that the text is suitable for further research. Fig. 6 illustrates the entire scheme of data acquisition, analysis and classification of posts.

The implementation of obtaining information may vary depending on the source of information, but maintain the general principle of its construction. The main purpose of the part of the software responsible for obtaining information from open sources is to perform actions quickly and efficiently. To achieve maximum performance, you must use the built-in methods for obtaining information from sources (API), if any. If there are no such methods, then it is necessary to obtain and extract the necessary information from HTTP requests.

There are three separate modules of the software package:

1. Information collection module - is responsible for receiving information from open sources and transmitting it for further processing; A large Python framework was built to parse data from VK social network. We used official VK API and partially parsed Kazakhstan profiles and stored data in Solr database;
2. Keyword search module - is responsible for finding keywords in a large amount of information; Since we already had a list of keywords that are often found in depressive posts, we implemented a linear search for words in each post, splitting it into tokens. Keywords for searching for potentially dangerous posts were prepared and validated by specialists from the republican scientific and practical centre of mental health;
3. Document ranking module - is responsible for determining whether the information is dangerous. To rank documents by the degree of danger were used word2vec vectorizer and deep learning algorithms such as Long Short Term Memory (LSTM) and Bidirectional Long Short Term Memory (BiLSTM).

The official documentation page of The Vkontakte social network contains basic information about the principles of the Vkontakte API [2]. API (application programming interface) is an intermediary between the application developer and the specific environment with which the application should interact. The API process simplifies code creation by providing a set of predefined classes, functions, or structures to work with existing data.

API “Vkontakte” is a ready-made interface that allows you to retrieve the necessary information from the database of the social network using https-requests to the server. The developer does not need to know how the database works in full details, and what tables it is made of – it is enough that the API request contains all the necessary data to access the server. The required query syntax and the type of data returned are defined on the service side.

Table 2 lists the components of a simple users query.get which as a request url looks like this ‘https://api.vk.com/method/users.get?user_id=210700286&v=5.92’

**Table 2:** Query component.

Component	Value
https://	Connection protocol.
api.vk.com/method	API service address.
User.get	Name of the API Vkontakte method.
?user_id=210700286&v=5.92	Query parameter.

Methods are conditional commands that correspond to a specific database operation. For example, users.get is the method to get users' information, account.getinfo returns information about the current user, etc.

All methods in the system are divided into sections. In the transmitted request you must pass the input data as GET parameters in the HTTP request after the method name. If the request is successfully processed, the server returns a JSON object with the requested data. The response structure for each method is strictly defined. The rules are specified on the pages describing the method in the official documentation.

In order to analyze the data, Python 3.7 programming language was applied with pandas, numpy, matplotlib, plotly, bokeh, cufflinks, spacy, googletrans packages as main libraries for calculation and visualization. Full description of programming code were given in Google Colaboratory [3] notebook by the https://colab.research.google.com/drive/1VSW3ofj8D6NEYziYazDcnSIIzAmw9mnA address [4]. Google Colaboratory required users to use google account for better exposition of graphs and figures.
